# Emotional distress following amyloid PET result disclosure: Heightened among those with cognitive symptoms

**DOI:** 10.1002/alz.70121

**Published:** 2025-04-06

**Authors:** Jeong Eun Kim, Dianxu Ren, Ann D. Cohen, Oscar L. Lopez, Jennifer H. Lingler

**Affiliations:** ^1^ Health & Community Systems University of Pittsburgh School of Nursing Pittsburgh Pennsylvania USA; ^2^ Department of Psychiatry University of Pittsburgh School of Medicine Pittsburgh Pennsylvania USA; ^3^ University of Pittsburgh Alzheimer's Disease Research Center Pittsburgh Pennsylvania USA; ^4^ Department of Neurology University of Pittsburgh School of Medicine Pittsburgh Pennsylvania USA

**Keywords:** Alzheimer's disease, amyloid, biomarker disclosure, dementia, disclosure‐related distress, mild cognitive impairment, psychological response

## Abstract

**INTRODUCTION:**

We examined differences in emotional distress following amyloid positron emission tomography (PET) results disclosure across the cognitive spectrum, including individuals who are cognitively unimpaired, with mild cognitive impairment (MCI), and dementia.

**METHODS:**

Seventy‐five participants from the University of Pittsburgh Alzheimer's Disease Research Center who completed baseline PET and an initial follow‐up call were included in the analysis. Multiple linear regression was employed to examine differences in distress among diagnostic groups, as measured by Impact of Genetic Testing for Alzheimer's Disease (IGT‐AD) adapted for amyloid PET.

**RESULTS:**

Regression analysis revealed significant associations between diagnostic categories and emotional distress post‐disclosure. Pairwise comparisons indicated that those with normal cognition experienced significantly lower distress compared to MCI and dementia groups, even after controlling for amyloid status.

**DISCUSSION:**

Emotional distress following amyloid PET disclosure is significantly higher in cognitively symptomatic individuals compared to the asymptomatic, highlighting the need for tailored counseling and support to address the complex psychological effects of disclosure.

**Highlights:**

- Emotional distress was compared across cognitive categories outside trial settings
- Cognitively unimpaired individuals had significantly lower distress than impaired
- No significant difference in distress was found between MCI and dementia groups
- Depression and anxiety did not differ significantly across diagnostic categories

## 1 INTRODUCTION

Recent developments in the field of Alzheimer's disease (AD) biomarker research have been paralleled by advances in our understanding of the nature and extent of psychological responses following disclosure of test results to patients and families. Studies have examined a range of populations, including cognitively unimpaired adults,^1^ individuals with subjective cognitive decline,^2^ and cognitively impaired adults^3^; and have mainly focused on the return of neuroimaging biomarkers, particularly amyloid positron emission tomography (PET). While receiving AD biomarker results can provide valuable insights into one's health status and facilitate informed decision‐making, it can also pose emotional challenges. These challenges often stem from concerns about worsening cognitive symptoms and the potential loss of independence.^4^

Current evidence indicates that receiving biomarker results typically does not lead to clinically significant increases in anxiety, depression, or suicidality. ^1^, ^2^, ^3^, ^5^, ^6^, ^7^ However, comparisons between amyloid‐positive and amyloid‐negative individuals generally find statistically significant difference by amyloid status, with relatively higher distress ratings among those testing positive.^1^, ^2^, ^3^, ^5^, ^6^, ^7^ Among cognitively unimpaired individuals, emotional distress has consistently been found to be low.^1^, ^5^, ^8^, ^9^, ^10^ There have been fewer studies with cognitively symptomatic individuals, and while distress levels are not typically severe, they may still be present, as evidenced by mild to moderately elevated scores on the Impact of Events Scale (IES), particularly among the amyloid positive.^3^

A recent systematic review highlighted that the infrequency of significant psychological distress among cognitively unimpaired samples includes amyloid positive persons, raising the question of whether post‐disclosure distress might vary based on whether or not the return of results occurs within the context of other potentially concerning factors like the presence of cognitive symptoms.^11^ However, it is unclear whether differences observed across studies are attributable to cognitive symptom status or are a result of differences in study methodologies.^11^ For example, many studies on amyloid PET disclosure in asymptomatic persons have been conducted within the context of clinical trials, where participants might be influenced by the prospect of receiving disease‐modifying treatments.^12^ This complicates the assessment of emotional distress and makes it challenging to compare results between symptomatic and asymptomatic participants.

This study aims to address these gaps by providing a direct comparison of emotional distress among cognitively unimpaired and cognitively impaired groups, specifically within a research setting that is independent of clinical trial screening. By doing so, it seeks to explore potential differences in the emotional impact of amyloid PET result disclosure across diagnostic categories, including cognitively normal individuals, those with mild cognitive impairment (MCI), and those with dementia.

## 2 METHODS

### 2.1 Sample and setting

The study sample was drawn from an ongoing brain imaging data repository study, PET Extension, which aims to obtain baseline PET imaging on a subset of University of Pittsburgh Alzheimer's Disease Research Center (ADRC) participants. Recruitment was conducted through the ADRC, and interested individuals were referred to the ADRC PET Extension research coordinator, who contacted them to discuss the informed consent process and schedule an appointment. The inclusion criteria to complete the PET scan were (1) be over 40 years of age, and (2) have completed a 3T research magnetic resonance imaging (MRI), including T1, T2 sequence within the past 36 months. The exclusion criteria were (1) inability to complete the PET scan due to claustrophobia, (2) inability to lie still for duration of scans, and (3) pregnancy or breastfeeding. In‐person consent for the PET scan was obtained by a study physician.

The method for determining amyloid status from the Pittsburgh compound B (PiB) PET scan has been previously described,^13^ and the cutoff scores were confirmed through visual assessment by a single expert nuclear medicine physician. Results were then available approximately 2 months post‐scan. Participants who requested their research results were required to sign an additional consent form acknowledging the psychological risks of learning their brain amyloid status and agreeing to follow‐up mood assessments. Additional exclusion criteria for result disclosure included (1) evidence of active, untreated primary psychiatric disorders (Center for Epidemiological Studies Depression score ≥ 16, or Spielberger State Anxiety score > 40); (2) active suicidal ideation indicated by Beck Scale for Suicidal Ideation score > 0, 2); (3) knowledge of amyloid status; and (4) in the opinion of the investigator, may cause significant psychological harm or otherwise negatively harm the participant.^14^, ^15^, ^16^

Eligibility for result disclosure was determined through screening assessments conducted by trained research team members. Screenings could be done via telephone, videoconferencing (Health Insurance Portability and Accountability Act [HIPAA]‐compliant Zoom), or in person at the ADRC, depending on participant preference. If participants were found eligible, they returned to the ADRC for written informed consent and an in‐person screening before the results feedback session. Screening assessments of mood and suicidal ideation were included, and if any scores were elevated, participants were contacted by a study psychologist or licensed counselor for follow‐up.

#### 2.1.1 Results feedback session and follow‐up assessments

A licensed physician on the study team conducted the results feedback session, which lasted approximately 30 min. This session could be conducted via telephone or videoconferencing, depending on the participant's preference and the physician's discretion. Although a guidance document was provided for the discussion, clinicians had the discretion to tailor the conversation as needed.

Follow‐up calls were conducted by a trained research team member via phone, with the goal of initial contact targeted for approximately 4 weeks after the disclosure. This assessment included the same mood and suicidality measures used during screening and the Impact of Genetic Testing for Alzheimer's Disease (IGT‐AD) scale adapted for amyloid PET.^3^, ^17^ If clinically significant symptoms of depression, anxiety, or suicidality were identified, participants were contacted by a licensed psychologist or counselor for additional support, with a second follow‐up call scheduled around 24 weeks post‐disclosure. Seventy‐five participants who underwent amyloid PET imaging and completed at least one follow‐up call were included in this analysis.

### 2.2 Study variables and instruments

Participants’ sociodemographic characteristics, including age, sex, race/ethnicity, educational attainment, and Mini‐Mental State Exam (MMSE) scores,^18^ were retrieved from ADRC records, where they are routinely collected as part of annual visits. The primary variable of interest was the level of emotional distress experienced after the disclosure of AD biomarker testing, measured using the IGT‐AD assessment,^17^ adapted for amyloid PET.^3^ The adapted IGT‐AD is a 16‐item questionnaire that assesses the emotional impact of disclosing amyloid PET results. Its focus on the unique emotional concerns associated with AD related testing makes it a valuable tool for understanding the psychological effects of biomarker disclosure compared to generalized mood scales or other event‐specific measures. The overall scale comprises two subscales: the distress subscale and the positive subscale. Participants rate their experiences on a four‐point scale (scored 0, 1, 3, or 5) over the past week, with higher scores indicating greater psychological distress. The positive subscale is scored reversely to maintain this interpretation. The IGT‐AD's external validity is supported by its positive correlation with other established measures of emotional distress, such as the Impact of Event Scale (IES), Beck Anxiety Inventory (BAI), and the Center for Epidemiologic Studies Depression Scale (CES‐D).^17^ The scale's internal reliability was confirmed with a Cronbach's alpha of 0.82.^17^

Depressive symptoms were assessed using the CES‐D, a 20‐item instrument rated on a four‐point scale.^14^ The CES‐D measures the frequency of depressive symptoms experienced during the preceding week, with scores ranging from 0 to 60, and a cut off score of 16 or higher.

State anxiety was measured using the Spielberger State Anxiety Inventory (STAI), a 20‐item, four‐point scale.^15^ Participants’ scores could range from 20 to 80, with higher scores indicating greater anxiety. A cutoff point of 40 has been suggested to detect clinically significant symptoms on the state anxiety scale.

**RESEARCH IN CONTEXT**

1. **Systematic review**: The authors reviewed the literature using traditional sources (e.g., PubMed). Relevant literature on psychological reactions to learning Alzheimer's disease (AD) biomarker results across cognitive categories (cognitively unimpaired, mild cognitive impairment, and dementia) are appropriately cited.
2. **Interpretation**: This study findings contribute to the literature by directly comparing emotional distress following amyloid positron emission tomography (PET) result disclosure across the cognitive categories under routine result return, highlighting the need for tailored communication strategies to address distress disparities between cognitively symptomatic and asymptomatic individuals.
3. **Future directions**: Future research should examine the psychological impact of biomarker‐informed AD diagnoses in a more culturally and socioeconomically diverse populations to better understand how these factors influence distress in the context of evolving biomarker‐driven treatments and diagnostic frameworks.

### 2.3 Statistical analysis

All analyses involving descriptive statistics and regression were performed using IBM SPSS Statistics for Mac (version 29, IBM, Corp.; Armonk, New York, USA). Descriptive statistics were employed to characterize the participants regarding their assigned sex at birth, age, race/ethnicity, educational attainment, MMSE scores, and their amyloid status. Comparisons between cognitively normal, MCI, dementia groups were performed using a one‐way analysis of variance (ANOVA) test, and *p*‐values less than 0.05 were considered statistically significant results. After screening, there were no missing data in this data analysis.

Multiple linear regression modeling was used to examine the relationship between emotional distress scores, and diagnostic categories, while controlling for participants’ sex, age, and amyloid status. Additional regression models were fitted to assess associations with other mood symptoms, including depression and anxiety. Pairwise comparisons were conducted to examine specific differences between diagnostic categories. Bonferroni correction was applied to adjust for multiple comparisons.

## 3 RESULTS

### 3.1 Descriptive statistics

Participants (*N* = 75) were predominantly white (*n* = 71), non‐Hispanic (*n* = 72) males (*n* = 44), with high educational attainment of average 16.4 years (standard deviation [SD] = 2.9). The average age of participants was 70.8 (SD = 8.5) years. The average MMSE scores for individuals who were cognitively normal was 28.6 (SD = 1.1), with MCI was 26.6 (SD = 2.3), and with dementia diagnosis 22.6 (SD = 4.3). The average number of days from disclosure to follow‐up data collection was 96.8 (SD = 61.4) days. Delays in data collection were due to difficulties in reaching some participants and staff unavailability for follow‐up calls.

During the initial follow‐up calls, participants’ mean CES‐D scores were 7.24 (SD = 7.40), and mean STAI scores were 28.64 (SD = 9.98) suggesting that, on average, participants did not exhibit persistent, clinically significant depressive or state anxiety symptoms in the first 3 months following disclosure. Detailed characteristics of the sample, categorized by cognitive status, are presented in Table 1. Although ANOVA revealed no significant differences in participants’ demographic characteristics across cognitive diagnostic categories, there were variations in the frequency of amyloid status among the groups.

**TABLE 1:** Characteristics of participants who are cognitively normal, with MCI, or dementia (*N* total = 75)

Characteristic	Cognitively normal (*n* = 21)	MCI (*n* = 24)	Dementia (*n* = 30)	*p*‐value (ANOVA)
Mean age in years (SD)	70.67 (7.75)	69.96 (8.25)	71.54 (9.28)	0.796
Education, *n* (%)^a^				
< High school	0 (0)	1 (4.2)	0 (0)	0.484
High school/GED	2 (9.5)	5 (20.8)	4 (13.3)	
Technical school or College	8 (38.1)	7 (29.2)	13 (43.3)	
Graduate school	11 (52.4)	11 (45.8)	13 (43.3)	
Sex, *n* (%)				
Male	9 (42.9)	14 (58.3)	21 (70.0)	0.157
Female	12 (57.1)	10 (41.7)	9 (30.0)	
Race/ethnicity, *n* (%)				
White/Caucasian	20 (95.2)	23 (95.8)	28 (93.3)	0.915
Black/African	1 (4.8)	1 (4.2)	2 (6.7)	
Hispanic, *n* (%)				
No	20 (95.2)	23 (95.8)	29 (96.7)	0.968
Yes	1 (4.8)	1 (4.2)	1 (3.3)	
Mean MMES score (SD)	28.6 (1.1)	26.6 (2.3)	22.6 (4.3)	n/a
Amyloid status, *n* (%)				
Elevated	6 (28.6)	13 (54.2)	19 (63.3)	0.046
Non‐elevated	15 (71.4)	11 (45.8)	11 (36.7)	
Mean IGT‐AD score (SD)	9.05 (9.79)	20.17 (16.28)	19.53 (14.34)	0.014
Mean CES‐D score (SD)	6.29 (7.36)	9.46 (9.05)	6.13 (5.61)	0.206
Mean STAI score (SD)	28.76 (10.48)	28.76 (9.37)	26.87(10.10)	0.369

Abbreviations: ANOVA, analysis of variance; CES‐D, Center for Epidemiologic Studies Depression Scale; GED, general educational development test; IGT‐AD, Impact of Genetic Testing for Alzheimer's Disease adapted for amyloid PET; MCI, mild cognitive impairment; MMSE, Mini‐Mental State Exam; SD, standard deviation; STAI, State‐Trait Anxiety Inventory.

^a^ Education was measured in years, however, for purposes of description, categorized.

### 3.2 Regression analysis and pairwise comparison

The findings from multiple linear regression model revealed significant association between participants’ diagnostic categories and their emotional distress scores measured by IGT‐AD post disclosure (b = 4.746, standard error [SE] = 2.066, *p* = 0.025) even after controlling for participants’ sex, age, and amyloid status. However, no significant associations were found for depressive symptoms, as measured by the CES‐D (b = 0.285, SE = 1.171, *p* = 0.808), or for state anxiety, as measured by the STAI (b = ‐1.022, SE = 1.570, *p* = 0.517).

Pairwise comparisons further indicated that individuals with normal cognition had significantly lower emotional distress compared to individuals with MCI (ΔM = ‐10.260, *p* = 0.045) and dementia (ΔM = ‐10.342, *p* = 0.044). No significant difference was found between MCI and dementia groups (ΔM = ‐0.083, *p* = 1.000). Additional details of the pairwise comparisons including the sex‐, age‐, and amyloid status‐adjusted mean scores of IGT‐AD, CES‐D, and STAI are presented in Table 2.

**TABLE 2:** Adjusted mean emotional distress scores across diagnostic categories, controlling for age, sex, and amyloid status, and results from multiple linear regression analysis

Outcomes	Adjusted mean (SE)			*p*‐value
	Cognitively normal	MCI	Dementia	
IGT‐AD	9.380 (3.065)	19.640 (2.685)	19.722 (2.488)	0.025^a^
CES‐D	5.473 (1.722)	9.550 (1.508)	6.629 (1.398)	0.808
STAI	28.651 (2.349)	30.598 (2.057)	27.066 (1.907)	0.517

*Note*: Adjusted means are estimated marginal means, controlling for age, sex, and amyloid status. *p*‐value less than 0.05 is considered statistically significant.

Abbreviations: CES‐D, Center for Epidemiologic Studies Depression Scale; IGT‐AD, Impact of Genetic Testing for Alzheimer's Disease adapted for amyloid PET; MCI, mild cognitive impairment; SE, standard error; STAI, State‐Trait Anxiety Inventory.

^a^ Pairwise comparisons revealed a significant difference in adjusted means between the cognitively normal group and both the MCI (*p* = 0.045) and dementia (*p* = 0.044) groups. However, no significant difference was found between the MCI and dementia groups (*p* = 1.000). *p*‐Values are adjusted for multiple comparisons using the Bonferroni correction.

## 4 DISCUSSION

Our findings reveal discernible differences in emotional distress levels between cognitively symptomatic and asymptomatic individuals within the first 3 months following amyloid PET result disclosure. Specifically, our analysis indicates that individuals with cognitive symptoms may experience higher distress than their asymptomatic counterparts. These findings align with existing literature showing that while anxiety, depression, and suicidality is not significantly heightened in either symptomatic or asymptomatic groups, disclosure‐related emotional distress is present, especially among symptomatic individuals with elevated amyloid PET results.^1^, ^2^, ^3^, ^19^ Importantly, this study is among the first to directly compare disclosure‐related distress across the cognitive spectrum under routine result return, outside of clinical trial screening settings.

Several factors likely explain varying levels of distress across diagnostic categories, including individuals’ understanding and interpretation of their biomarker results. In a qualitative study by Largent and colleagues,^20^ cognitively unimpaired clinical trial screening candidates perceived amyloid imaging results as affecting their sense of self and posing potential social consequences, such as stigma and discrimination in employment, healthcare, or insurance. For these individuals, an elevated amyloid PET results raised concerns beyond immediate symptoms, including future decline and its social ramifications. Despite facing the challenges noted by Largent and colleague's qualitative analysis, individuals who are cognitively normal may still plausibly hope to not develop dementia, which could explain the lower levels of distress compared to the cognitively symptomatic individuals.^20^

In a separate study by James and colleagues,^21^ patients with MCI or early‐stage dementia, along with their care partners, struggled to interpret an elevated amyloid PET result due to confusion regarding terminology and perceived uncertainty regarding implications for disease progression. Such lack of clarity could contribute to emotional burden. For individuals with MCI, amyloid positivity may shift concerns from an uncertain risk of progression to a more concrete expectation of worsening symptoms. Similarly, for individuals already diagnosed with dementia, an elevated amyloid PET result may solidify their diagnosis, intensifying distress by reinforcing the irreversible nature of their condition. Notably, Lingler and colleagues^3^ found that perceived ambiguity regarding MCI actually increased in patients with non‐elevated amyloid results, as these individuals were aware their symptoms remained unexplained. This highlights the need for clear communication to help patients understand both the limitations and implications of biomarker results, including those receiving negative results.

The current findings come at a pivotal moment in the discourse of AD diagnostics. While there is a lack of substantial empirical evidence for severe psychological harm following AD biomarker disclosure, concerns about such harm are resurfacing in light of proposals to further integrate biomarkers into diagnostic criteria for AD. Critics of the recent framework proposed by Jack and colleagues^22^ caution that biomarker results might be interpreted not only by researchers and specialists, but also by patients as definitive indicators of AD dementia, rather than as markers of risk or explanations of symptom etiology. While this framing could potentially amplify distress, additional research is needed to understand whether a biomarker‐informed AD diagnosis introduces psychological distress above and beyond what is experienced when receiving a syndromally based dementia diagnosis.

Our study also contributes to an understanding of how evolving treatment options may influence patients’ reactions to biomarker disclosure. Unlike prior studies that largely predate the advent of amyloid‐lowering therapies, our findings may have relevance for patient distress in anticipation of therapeutic decisions post‐disclosure. With blood‐based biomarkers becoming more prevalent and new therapeutic options emerging, biomarker results carry increasingly complex implications, underscoring the need for tailored support systems addressing patients’ psychological and practical concerns.

However, certain limitations should be considered. Notably, our sample was highly homogeneous in sociodemographic characteristics, limiting the generalizability of results to more culturally and economically diverse populations, as emotional responses to AD testing may be influenced by sociocultural factors, as seen in varying levels of interest in AD testing and diverse reactions to results across cultural backgrounds.^23^, ^24^ Additionally, the time between disclosure and follow‐up data collection varied among participants. However, ANOVA revealed no significant differences in the time to data collection from result disclosure across cognitive diagnostic categories (F(2, 72) = 1.913, *p* = 0.155). It also bears mention that documenting distress within the first 3 months of disclosure provides important insights into the phenomenon of persistent distress that may warrant clinical intervention. Lastly, there is no established cutoff score for the IGT‐AD, rendering score interpretation somewhat ambiguous. Future research should aim to establish guidelines for interpreting IGT‐AD scores to enhance this tool's clinical utility.

Overall, our findings have important implications for counseling and support in the context of AD biomarker disclosure. Integrating appropriate counseling while addressing the broader significance of AD biomarker disclosure on patients’ lives, including issues related to identity, stigma, and life planning, as these factors significantly influence well‐being and quality of life post‐disclosure.^25^ As AD research advances, developing comprehensive support systems that address patients' multifaceted needs will be critical to ensuring biomarker disclosure promotes psychological well‐being and informed decision‐making.

## CONFLICT OF INTEREST STATEMENT

Dr. Jennifer Lingler has provided paid consultation to Biogen and Genentech. All other authors have no conflicts of interest to report. Author disclosures are available in the supporting information.

## Supporting information



Supporting Information

## References

[1] Grill JD , Raman R , Ernstrom K , et al. A4 Study Team. Short‐term psychological outcomes of disclosing amyloid imaging results to research participants who do not have cognitive impairment. JAMA Neurol. 2020;77(12):1504‐1513. doi:10.1001/jamaneurol.2020.2734 32777010 PMC7418046

[2] Caprioglio C , Ribaldi F , Visser LNC , et al. AMYPAD consortium. Analysis of psychological symptoms following disclosure of amyloid‐positron emission tomography imaging results to adults with subjective cognitive decline. JAMA Netw Open. 2023;6(1):e2250921. doi:10.1001/jamanetworkopen.2022.50921 36637820 PMC9857261

[3] Lingler JH , Sereika SM , Butters MA , et al. A randomized controlled trial of amyloid positron emission tomography results disclosure in mild cognitive impairment. Alzheimers Dement. 2020;16(9):1330‐1337. doi:10.1002/alz.12129 32588971 PMC7541680

[4] Vanderschaeghe G , Schaeverbeke J , Bruffaerts R , Vandenberghe R , Dierickx K . Amnestic MCI patients' experiences after disclosure of their amyloid PET result in a research context. Alzheimers Res Ther. 2017;9(1):92. doi:10.1186/s13195-017-0321-3 29197423 PMC5712105

[5] Burns JM , Johnson DK , Liebmann EP , Bothwell RJ , Morris JK , Vidoni ED . Safety of disclosing amyloid status in cognitively normal older adults. Alzheimer Dement. 2017;13(9):1024‐1030. doi:10.1016/j.jalz.2017.01.022 PMC5582024 28263740

[6] Lim YY , Maruff P , Getter C , Snyder PJ . Disclosure of positron emission tomography amyloid imaging results: a preliminary study of safety and tolerability. Alzheimer Dement. 2016;12(4):454‐458. doi:10.1016/j.jalz.2015.09.005 26750717

[7] de Wilde A , van Buchem MM , Otten RHJ , et al. Disclosure of amyloid positron emission tomography results to individuals without dementia: a systematic review. Alzheimer Res Ther. 2018;10(1):72. doi:10.1186/s13195-018-0398-3 PMC6064628 30055660

[8] Carpenter BD , Xiong C , Porensky EK , et al. Reaction to a dementia diagnosis in individuals with Alzheimer's disease and mild cognitive impairment. J Am Geriatr Soc. 2008;56(3):405‐412. doi:10.1111/j.1532-5415.2007.01600.x 18194228

[9] Hendriksen HMA , de Rijke TJ , Fruijtier A , et al. Amyloid PET disclosure in subjective cognitive decline: patient experiences over time. Alzheimer Dement. 2024;20(9):6556‐6565. doi:10.1002/alz.14148 PMC11497681 39087383

[10] Wake T , Tabuchi H , Funaki K , et al. Disclosure of amyloid status for risk of Alzheimer disease to cognitively normal research participants with subjective cognitive decline: a longitudinal study. Am J Alzheimer Dis Dement. 2020;35:1533317520904551. doi:10.1177/1533317520904551 PMC10623980 32052640

[11] Couch E , Ashford MT , Zhang W , Prina M . Psychosocial and behavioral outcomes for persons with cognitive impairment and caregivers following amyloid‐β PET scan disclosure: a systematic review. Alzheimer Dis Assoc Disord. 2023;37(3):246‐258. doi:10.1097/WAD.0000000000000569 37561950 PMC10529389

[12] Kim H , Lingler JH . Disclosure of amyloid PET scan results: a systematic review. Progr Mol Biol Trans Sci. 2019;165:401‐414. doi:10.1016/bs.pmbts.2019.05.002 31481171

[13] Cohen AD , Mowrey W , Weissfeld LA , et al. Classification of amyloid‐positivity in controls: comparison of visual read and quantitative approaches. NeuroImage. 2013;71:207‐215. doi:10.1016/j.neuroimage.2013.01.015 23353602 PMC3605888

[14] Radloff L . The CES‐D scale: a self‐report depression scale for research in the general population. Appl Psychol Measure. 1977;1(3):385‐401.

[15] Spielberger CD . In: Spielberger CD , Sarason EG , eds. Anxiety: state‐trait process. In *Stress and Anxiety* . Hemisphere Publishing Corp; 1975.

[16] Beck AT , Kovacs M , Weissman A . Assessment of suicidal intention: the scale for suicide ideation. J Consult Clin Psychol. 1979;47(2):343‐352. doi:10.1037//0022-006x.47.2.343 469082

[17] Chung WW , Chen CA , Cupples LA , et al. A new scale measuring psychologic impact of genetic susceptibility testing for Alzheimer disease. Alzheimer Dis Assoc Disord. 2009;23(1):50‐56. doi:10.1097/wad.0b013e318188429e 19266699 PMC2743905

[18] Folstein MF , Folstein SE , McHugh PR . “Mini‐mental state”. A practical method for grading the cognitive state of patients for the clinician. J Psychiatr Res. 1975;12(3):189‐198. doi:10.1016/0022-3956(75)90026-6 1202204

[19] Clark LR , Erickson CM , Chin NA , et al. Psychosocial implications of learning amyloid PET results in an observational cohort. Alzheimers Dement. 2024;20(9):6579‐6589. doi:10.1002/alz.14153 39129396 PMC11497643

[20] Largent EA , Harkins K , van Dyck CH , Hachey S , Sankar P , Karlawish J . Cognitively unimpaired adults' reactions to disclosure of amyloid PET scan results. PLoS One. 2020;15(2):e0229137. doi:10.1371/journal.pone.0229137 32053667 PMC7018056

[21] James HJ , Van Houtven CH , Lippmann S , et al. How accurately do patients and their care partners report results of amyloid‐β PET scans for Alzheimer's disease assessment?. J Alzheimers Dis. 2020;74(2):625‐636. doi:10.3233/JAD-190922 32065790 PMC7183243

[22] Jack CR Jr , Andrews JS , Beach TG , et al. Revised criteria for diagnosis and staging of Alzheimer's disease: Alzheimer's Association Workgroup. Alzheimers Dement. 2024;20(8):5143‐5169. doi:10.1002/alz.13859 38934362 PMC11350039

[23] Wikler EM , Blendon RJ , Benson JM . Would you want to know? Public attitudes on early diagnostic testing for Alzheimer's disease. Alzheimers Res Ther. 2013;5(5):43. doi:10.1186/alzrt206 24010759 PMC3978817

[24] Ritchie M , Salazar CR , Gillen DL , Grill JD . Post‐disclosure distress among racial and ethnic groups in a preclinical AD trial. Alzheimers Dement. 2024;20(4):2508‐2515. doi:10.1002/alz.13726 38329007 PMC11032552

[25] Wang Y , Ren D , Roberts JS , Tamres LK , Lingler JH . Value of knowing: health‐related behavior changes following amyloid PET results disclosure in mild cognitive impairment. J Prev Alzheimers Dis. 2024;11(4):958‐965. doi:10.14283/jpad.2024.50 39044506

